# Further Insights into the Toxicity of *Bacillus cytotoxicus* Based on Toxin Gene Profiling and Vero Cell Cytotoxicity Assays

**DOI:** 10.3390/toxins13040234

**Published:** 2021-03-24

**Authors:** Johanna Burtscher, Danai Etter, Michael Biggel, Janine Schlaepfer, Sophia Johler

**Affiliations:** 1 Institute of Food Science, BOKU—University of Natural Resources and Life Sciences Vienna, 1190 Vienna, Austria; 2 Institute for Food Safety and Hygiene, Vetsuisse Faculty, University of Zürich, 8057 Zurich, Switzerland; danai.etter@uzh.ch (D.E.); michael.biggel@uzh.ch (M.B.); janine.schlaepfer@uzh.ch (J.S.); sophia.johler@uzh.ch (S.J.)

**Keywords:** *cytK*, enterotoxins, Vero cell assay, mashed potato powder, *nhe* variant

## Abstract

*Bacillus cytotoxicus* belongs to the *Bacillus cereus* group that also comprises the foodborne pathogen *Bacillus cereus* sensu stricto, *Bacillus anthracis* causing anthrax, as well as the biopesticide *Bacillus thuringiensis*. The first *B. cytotoxicus* was isolated in the context of a severe food poisoning outbreak leading to fatal cases of diarrheal disease. Subsequent characterization of the outbreak strain led to the conclusion that this *Bacillus* strain was highly cytotoxic and eventually resulted in the description of a novel species, whose name reflects the observed toxicity: *B. cytotoxicus*. However, only a few isolates of this species have been characterized with regard to their cytotoxic potential and the role of *B. cytotoxicus* as a causative agent of food poisoning remains largely unclear. Hence, the aim of this study was to gain further insights into the toxicity of *B. cytotoxicus*. To this end, 19 isolates were obtained from mashed potato powders and characterized by toxin gene profiling and Vero cell cytotoxicity assays. All isolates harbored the *cytK1* (cytotoxin K1) gene and species-specific variants of the *nhe* (non-hemolytic enterotoxin) gene. The isolates exhibited low or no toxicity towards Vero cells. Thus, this study indicates that the cytotoxic potential of *B. cytotoxicus* may be potentially lower than initially assumed.

## 1. Introduction

*B. cytotoxicus* belongs to the *B. cereus* group. This group of endospore-forming bacteria comprises several closely related species that differ significantly in toxicity and pathogenicity potential including probiotic strains, plant growth promoters and biopesticides as well as highly toxic strains, occasionally causing fatalities [1,2]. Several members of the *B. cereus* group have been linked to emetic or diarrheal foodborne illnesses. The causative agents of gastrointestinal symptoms are different toxins. The intake of the heat-stable toxin cereulide, which is encoded by the *ces* locus, causes emesis and nausea and its presence is attributed to the growth of emetic *B. cereus* in food [2]. Heat-labile toxins, in contrast, such as non-hemolytic enterotoxins (Nhe), hemolysin BL (Hbl), and cytotoxin K (CytK), lead to diarrheal toxico-infections, as toxin production occurs in the small intestine [2,3]. *B. cytotoxicus* is known to cause diarrheal symptoms and has not been linked to emetic illness. It represents a relatively homogenous species, which is clearly distinguishable from other members of the *B. cereus* group [4,5]. The first documented isolation of *B. cytotoxicus* from vegetable puree took place in 1998 in France during a severe food poisoning outbreak that resulted in 44 ill individuals, including three fatalities [6]. Since then, further cases of food poisoning associated with vegetable purees have been described [7,8]. However, at the time of sampling, the isolates were classified as *B. cereus* and only later assigned to *B. cytotoxicus* after the introduction of the novel species in 2013 [7]. *B. cytotoxicus* is characterized by its thermotolerance, which seems to confer a growth advantage in processed foods, such as vegetable purees. Furthermore, the absence of starch hydrolysis, the dependency on tryptophan for growth, and the presence of a specific *cytK1* variant of the gene encoding cytotoxin K are typical attributes of this species [7]. CytK1 is one of two known genetic variants of cytotoxin K, a pore-forming toxin with necrotic and hemolytic activity [9]. Fagerlund, et al. [10] observed higher toxicity towards human intestinal CaCo-2 cells and Vero cells for CytK1 than for CytK2, which is produced by other members of the *B. cereus* group. As a consequence, they concluded that the high cytotoxic activity of CytK1 may have caused the high virulence of the *B. cytotoxicus* outbreak strain from France. However, present data on the cytotoxicity of this species are ambiguous and indicate strain-specific differences. While the high toxicity of strains associated with food poisoning incidents was mostly confirmed [9], studies using randomly isolated strains from food yielded varying results. For instance, Vero cell cytotoxicity assays testing isolates from powdered mashed potato products revealed low or no cytotoxicity for eight strains, thus indicating that the toxic potential of *B. cytotoxicus* may have been overestimated [11]. In contrast, one isolate exhibited exceptionally high toxicity, significantly surpassing the toxicity of a *B. cereus* sensu lato outbreak strain that was used as a reference [11]. To our knowledge, the cytotoxicity data of *B. cytotoxicus* are currently limited to 12 isolates. Therefore, further data are pivotal to assess the health risk originating from the presence of *B. cytotoxicus* in food. Hence, within this study, we aimed to sharpen our understanding of the cytotoxic potential of this species. To this end, we isolated *B. cytotoxicus* strains from mashed potato powders in order to characterize their toxin genes and their cytotoxic potential.

## 2. Results and Discussion

### 2.1. Prevalence in Mashed Potato Powders

We detected at least one isolate of *B. cereus* sensu lato in each tested brand of mashed potato powder and found *B. cytotoxicus* in 19 out of the 20 (95%) purchased samples (see Table 1). The high prevalence of *B. cytotoxicus* in mashed potato powders revealed within this study is in good agreement with the results of previous studies on mashed potatoes, other heat-treated potato products, or products made thereof [11,12,13,14]. The ecological niche of *B. cytotoxicus* and the contamination pathway of potato products, however, remain unclear. Indeed, the first isolation of this species from a natural habitat (geothermal areas) was published only recently, but it does not provide any indication of potential food contamination pathways [15]. Stevens, et al. [4] revealed that *B. cytotoxicus* harbors a putative hydroxyphenylalanine (*hpa*) operon, which is absent in the genomes of other *B. cereus* group members. They concluded that the presence of this operon may confer an evolutionary advantage to *B. cytotoxicus* as it enables the species to metabolize aromatic compounds and to potentially inhibit Gram-negatives in the intestine by producing the inhibiting agent para-cresol. Furthermore, *hpa*-encoded pathways are often associated with soil bacteria, thus indicating soil as the ecological niche of *B. cytotoxicus* [4]. Moreover, it can be assumed that the heat treatment applied during the production of dried potato products also provides a selective advantage to the thermotolerant *B. cytotoxicus*. In addition, the growth of *B. cytotoxicus* in mashed potatoes has been reported after keeping cooked puree at room temperature for several hours [12]. This is particularly relevant because vegetable purees are often served in mass catering facilities, where temperature control during food handling is crucial, albeit challenging. The risk of *B. cytotoxicus* intoxications associated with mass catering facilities is also reflected in epidemiological data. For instance, all five registered foodborne outbreaks in France from 2007 to 2014 due to *B. cytotoxicus* happened in school or staff canteens, retirement homes, or community facilities [16]. However, consideration must be given to the fact that *B. cytotoxicus* as a causative agent of foodborne illness may be underreported in other settings due to the generally rather mild and self-limiting symptoms that did not require medical attention or further microbiological tests [8]. Moreover, in the case of microbiological food analyses, members of the *B. cereus* group are not usually further differentiated, which partly explains the limited amount of data at the species level [17]. Therefore, it is all the more important to add cytotoxicity data to the body of knowledge, thus improving our understanding of the toxic potential of *B. cytotoxicus* in food.

### 2.2. Toxin Gene Profiling

The 19 isolates recovered from different mashed potato powder samples of seven distinct brands showed homogenous toxin profiles (Table 1). Each isolate harbored the gene *cytK1*, whereas the genes *cytK2*, *hbl*, and *ces* were not detected. This toxin profile in *B. cytotoxicus* is consistent with previous studies. So far, no *B. cytotoxicus* strains have been discovered that harbor the *ces* gene encoding cereulide and the *cytK1* variant of the *cytK* gene has been determined as a characteristic feature of *B. cytotoxicus* [7,11,16]. Cytotoxin K is a pore-forming, necrotic, and hemolytic protein that was initially isolated from an outbreak strain that had been linked to severe food poisoning [6]. The high virulence of this outbreak strain was at least partly attributed to the greater cytotoxic activity of CytK1 towards Vero cells compared to CytK2 [10]. However, the later discovery of non-cytotoxic strains harboring the *cytK1* gene led to the assumption that the detection of this gene variant does not necessarily indicate highly cytotoxic strains [9,18].

It is known that while all *B. cereus* sensu lato harbor *nhe*, classical *nhe* primers fail to detect *nhe* in *B. cytotoxicus*. The nhe_Bcyt_ primers used in this study were designed and validated based on all publicly available and whole genome sequences of *B. cytotoxicus*. In preliminary PCR runs using the new primers, the presence of the *nhe* gene variant of *B. cytotoxicus* was confirmed in 13 additional *B. cytotoxicus* isolates from the internal culture collection (data not shown). The *nhe* gene variant of the reference strain *B. cereus* sensu stricto was not amplified (see Table 1).

Across the tested strain set within this study, the *nhe* gene encoding the non-hemolytic enterotoxin complex Nhe was not detected using standard primers targeting the *nhe* gene of *B. cereus* sensu lato [19]. However, all tested strains were positive for the *nhe* gene using primers (nhe_Bcyt_). This is consistent with the finding that the *nhe* gene variant of *B. cytotoxicus* yields only about 80% identity in protein sequence compared with the previously described Nhe toxins in *B. cereus* [9]. The presence of the *nhe* operon in *B. cytotoxicus* also corroborates previous observations that nearly 100% of enteropathogenic *B. cereus* strains harbor this gene [8,20]. The primers described within this study appear to be a useful tool allowing for *nhe* screening in *B. cytotoxicus* in future studies. However, although the presence of enterotoxin genes, such as *cytK1* and *nhe*, may indicate potential toxic effects, it is well known that the toxic activity of a strain cannot be derived from the sole presence of enterotoxin genes [9,11]. Indeed, toxin production is a complex process that requires the coordinated action of several factors, including transcriptional, posttranscriptional and posttranslational regulatory mechanisms [21].

### 2.3. Vero Cell Cytotoxicity

A Vero cell assay was performed to investigate a potential cytotoxic impact. Vero cells were used due to the wide range of experience in testing *B. cereus* cytotoxicity using this cell type and a higher susceptibility of Vero cells towards Nhe than towards Hbl, which has hitherto seemed to be irrelevant in *B. cytotoxicus*. Indeed, Nhe seems to explain most of the cytotoxic potential in Vero cell assays and appears to be a good indicator for the diarrheic potential of *B. cereus* because food poisoning strains tend to produce high levels of this enterotoxin [20].

Reciprocal titers obtained in the Vero cell assay are shown in Table 1. No cytotoxic effect was observed for eight of the *B. cytotoxicus* strains (CH 301, CH 302, CH 306, CH 307, CH 309, CH 310, CH 311, CH 312). Eleven strains exhibited cytotoxic effects, albeit all strains are considered to be low-level enterotoxin producers. This outcome supports the assumption of Heini et al. [11]—that most *B. cytotoxicus* strains isolated from food seem to be non-toxic with a few highly cytotoxic exceptions—as well as the hypothesis of Fagerlund, et al. [9]—that the cytotoxicity of this species may have been overestimated. As information about the cytotoxicity of *B. cytotoxicus* is still extremely scarce, the current study adds valuable Vero cytotoxicity data to the current state of knowledge.

It has recently become evident that several other parameters may contribute to food toxico-infections caused by *B. cereus* sensu lato. This includes consumed foodstuff and stomach passage as well as strain-specific characteristics and virulence factors, such as; spore germination, motility, adhesion, and secreted enzymes [2,8,22,23]. Further research into the role of these factors and the hazardous potential of a strain or meal is crucial for risk assessment.

## 3. Conclusions

In conclusion, this study provides further insights into the cytotoxicity of the rarely explored species *B. cytotoxicus*. We observed a high prevalence of *B. cytotoxicus* in mashed potato powders and demonstrated that a majority of isolates show no or low cytotoxicity towards Vero cells, despite the presence of enterotoxin genes *cytK1* and *nhe*. This corroborates previous assumptions that the hazardous potential of this species may be lower than initially assumed. Nevertheless, it has to be kept in mind that the thermotolerant spore former *B. cytotoxicus* can endure the production process of dehydrated products and is able to grow into hazardous concentrations when these products are held at improper temperatures. The results of this study contribute towards a better understanding and an improved risk assessment of *B. cytotoxicus*.

## 4. Materials and Methods

### 4.1. Isolation of B. cytotoxicus from Mashed Potato Powder

A total of 20 mashed potato powders from 7 different brands (A–G) had been purchased from Swiss supermarkets in 2018 and 2020 (see Table 1). Two isolation procedures were performed in parallel and in accordance with the protocols described by Heini, et al. [11] and Contzen, et al. [14]. These protocols included different media for enrichment (buffered peptone water (Oxoid, Basel, CH) and CGY Medium [24]), streaking (Mossel agar and sheep blood agar), and different incubation temperatures (37 and 46 °C). Characteristic colonies (rough and dry showing hemolysis on blood agar or rough and dry with a pink background surrounded by an egg yolk precipitate on Mossel agar) were selected and subcultured in CGY broth. Pure cultures were stored at −80 °C after the addition of 20% (*v*/*v*) glycerol.

### 4.2. DNA Extraction and Toxin Gene Profiling

Prior to DNA extraction, presumptive *B. cytotoxicus* strains were reactivated by streaking them on sheep blood or Mossel agar. Then, single colonies were chosen to prepare overnight cultures in CGY broth. After incubation at 37 °C, DNA was extracted using the DNeasy Blood and Tissue Kit (Qiagen, Hombrechtikon, Switzerland). Obtained isolates were screened for the presence of *nhe* (non-hemolytic enterotoxin), *cytK* (cytotoxin K), *ces* (cereulide) and *hbl* (hemolysin BL) genes in a multiplex PCR assay described by Ehling-Schulz et al. [19]. Further discrimination between *cytK1* and *cytK2* was achieved using the primers published by Guinebretiere et al. [25]. Because sequence analysis of publicly available genomes showed that the *nhe* gene of *B. cytotoxicus* differs from other members of the *B. cereus* group, a primer pair were designed to specifically target the *nhe* operon of *B. cytotoxicus*. The primer nhe_BcytF_ (5′-CGG AAG CAG AGG TAA CAG AAA-3′) binds to the *nhe A* gene and the primer nhe_BcytR_ (5′- GTG GCT ACA GAG GAA CCA ATAA-3′) binds to the *nhe B* gene, yielding an amplicon length of 1045 bp. For all PCR reactions, the GoTaq G2 Hot Start master mix (Promega, Madison, USA) was used with the following standard PCR program: initial denaturation at 95 °C for 2 min, 30 cycles of denaturation at 95 °C for 45 s, annealing at 51 °C for 45 s, and extension at 72 °C for 2 min, followed by a final extension of 5 min at 72 °C. If necessary, annealing temperatures were adjusted to the primer system and the extension time was adapted to the expected amplicon length.

### 4.3. Vero Cell Cytotoxicity Assay

Cell-free supernatants were produced using CGY broth. First, an overnight culture of each *B. cytotoxicus* strain was prepared in 3 mL of CGY broth (incubation at 30 °C, shaking at 150 rpm). Grown cultures were then inoculated into 30 mL of CGY in an Erlenmeyer flask at an OD_600_ of 0.05 and incubated at 30 °C (shaking at 150 rpm) until an OD_600_ of 7 was reached. Subsequently, bacterial biomass was removed by centrifuging 5 mL of the culture at 11,000 rpm for 10 min at 4 °C and by passing the obtained supernatant through a sterile filter (0.2 µm pore size). Aliquots of 1 mL sterile supernatant were supplemented with 10 µL of 0.1 M EDTA-Na_2_ and stored at −80 °C. The cytotoxicity of the supernatants was assessed in a Vero cell assay using WST-1 as described by Moravek, et al. [20]. In brief, serial dilutions of the supernatants were distributed into microtiter plates containing Vero cell suspensions (10^4^ cells per well) in Dulbecco’s modified Eagle medium supplemented with 1 % fetal calf serum and 0.4 % penicillin-streptomycin (5000 U/mL). After incubation for 24 h at 37 °C and 5% CO_2_, the extinction of the produced formazan dye from WST-1 was measured at 450 nm in order to determine the mitochondrial activity of viable cells. The obtained data were transformed into a dose–response curve, which was used to calculate the 50% loss of mitochondrial activity, shown as reciprocal cytotoxicity titers. For each strain, two independent Vero cell assays were performed, each including two technical replicates of each dilution series per strain. The food poisoning *B. cereus* sensu stricto strain NVH 0075-95 was used as a highly cytotoxic reference strain. Strains were classified into toxicity levels according to Jeßberger, et al. [21] and cutoffs were normalized based on the mean of the reference strain according to Johler, et al. [1]. Absolute cytotoxicity values <0.4 were considered as low-level, values from 0.4 to 0.8 were considered as mid-level, and values >0.8 were considered as high-level enterotoxin producers.

## Figures and Tables

**Table 1:** Overview of toxin genes detected by PCR (polymerase chain reaction) in strains isolated from mashed potato powders (sample numbers 1–20) of brands A–G and results of a WST-1 bioassay using Vero cells shown as normalized reciprocal cytotoxicity titers.

				Toxin Genes					Enterotoxin Production in Vero Cell Assay	
Sample Number	Strain ID	Species	Brand	*nhe* ^1^	*nhe*_Bcyt_	*hbl*	*cytK*	*ces*	Normalized Reciprocal Titer ^2^	Standard Deviation ^3^
1	CH_301	*B. cytotoxicus*	A	-	+	-	+ (*cytK1*)	-	0.00	0.00
2	CH_302	*B. cytotoxicus*	B	-	+	-	+ (*cytK1*)	-	0.00	0.00
3	CH_303	*B. cytotoxicus*	A	-	+	-	+ (*cytK1*)	-	0.03	0.03
4	CH_304	*B. cytotoxicus*	C	-	+	-	+ (*cytK1*)	-	0.03	0.03
5	CH_305	*B. cytotoxicus*	D	-	+	-	+ (*cytK1*)	-	0.15	0.15
6	CH_306	*B. cytotoxicus*	B	-	+	-	+ (*cytK1*)	-	0.00	0.00
7	CH_307	*B. cytotoxicus*	B	-	+	-	+ (*cytK1*)	-	0.00	0.00
8	CH_308	*B. cytotoxicus*	D	-	+	-	+ (*cytK1*)	-	0.07	0.07
9	CH_309	*B. cytotoxicus*	C	-	+	-	+ (*cytK1*)	-	0.00	0.00
10	CH_310	*B. cytotoxicus*	E	-	+	-	+ (*cytK1*)	-	0.00	0.00
11	CH_311	*B. cytotoxicus*	A	-	+	-	+ (*cytK1*)	-	0.00	0.00
12	CH_312	*B. cytotoxicus*	F	-	+	-	+ (*cytK1*)	-	0.00	0.00
13	CH_313	*B. cytotoxicus*	F	-	+	-	+ (*cytK1*)	-	0.01	0.01
14	CH_314	*B. cytotoxicus*	C	-	+	-	+ (*cytK1*)	-	0.01	0.01
15	CH_315	*B. cytotoxicus*	B	-	+	-	+ (*cytK1*)	-	0.02	0.02
16	CH_316	*B. cytotoxicus*	A	-	+	-	+ (*cytK1*)	-	0.08	0.06
17	CH_317	*B. cytotoxicus*	E	-	+	-	+ (*cytK1*)	-	0.03	0.03
18	CH_318	*B. cytotoxicus*	G	-	+	-	+ (*cytK1*)	-	0.09	0.09
19	CH_319	*B. cereus* sensu lato	B	+	-	-	-	-	n.a.	n.a.
20	CH_647	*B. cytotoxicus*	D	-	+	-	+ (*cytK1*)	-	0.32	0.09
n.a.	NVH 0075-95^2^	*B. cereus* sensu stricto	n.a.	+	-	-	-	-	1.00	0.63

+ positive, - negative, n.a. not available. ^1^ As the *nhe* gene variant of *B. cytotoxicus* was not detected using standard primers targeting the *nhe* gene of *B. cereus* sensu lato, primers specifically detecting the *nhe* variant occurring in *B. cytotoxicus* strains were used (nhe_Bcyt_). ^2^ Reciprocal titers are provided as absolute values normalized using the absolute value of the highly cytotoxic *B. cereus* sensu stricto reference strain NVH 0075-95 of the same Vero cell run. ^3^ Two independent Vero cell runs were performed, each including two technical replicates of each dilution series per *Bacillus* strain.

## Data Availability

Not applicable.
